# Health Behaviors, Care Needs and Attitudes towards Self-Prescription: A Cross-Sectional Survey among Dutch Medical Students

**DOI:** 10.1371/journal.pone.0028038

**Published:** 2011-11-21

**Authors:** Tjeerd Van der Veer, Monique H. W. Frings-Dresen, Judith K. Sluiter

**Affiliations:** Coronel Institute of Occupational Health, Academic Medical Center (AMC), Amsterdam, The Netherlands; Finnish Institute of Occupational Health, Finland

## Abstract

**Purpose:**

There is a growing awareness of the potent ways in which the wellbeing of physicians impacts the health of their patients. The purpose of this study was to investigate the health behaviors, care needs and attitudes towards self-prescription of Dutch medical students, and any differences between junior preclinical and senior clinically active students.

**Methods:**

All students (n = 2695) of a major Dutch medical school were invited for an online survey. Physical activity, eating habits, alcohol consumption, smoking, Body Mass Index, substance use and amount of sleep per night were inquired, as well as their need for different forms of care and their attitude towards self-prescription.

**Results:**

Data of 902 students were used. Physical activity levels (90% sufficient) and smoking prevalence (94% non-smokers) were satisfying. Healthy eating habits (51% insufficient) and alcohol consumption (46% excessive) were worrying. Body Mass Indexes were acceptable (20% unhealthy). We found no significant differences in health behaviors between preclinical and clinically active students. Care needs were significantly lower among clinically active students. (p<0.05) Student acceptance of self-prescription was significantly higher among clinically active students. (p<0.001)

**Conclusions:**

Unhealthy behaviors are prevalent among medical students, but are no more prevalent during the clinical study phase. The need for specific forms of care appears lower with study progression. This could be worrying as the acceptance of self-care and self-prescription is higher among senior clinical students. Medical faculties need to address students' unhealthy behaviors and meet their care needs for the benefit of both the future physicians as well as their patients.

## Introduction

Over the past few years, a great deal of research has been done on the relationship between doctors' and medical students' own health and how this affects the health care of their patients. It has become clear that healthy physicians set a strong example for their patients and have an improved ability to motivate their patients to change unhealthy behaviors [1]–[4]. The same has been proven for medical students: their personal healthy habits influence their counseling practices in a positive way, for physical activity [5], as well as for smoking and drinking [6], [7]. These findings suggest that in promoting health in our medical students, we have found a novel approach to promote health in the general population.

Yet, from experience we know that students' health is hardly addressed in medical school and that the medical training is demanding and could even cause the student to neglect healthy habits. Aspects of medical students' health have been monitored in several countries, with results showing that although the medical students are generally healthier than their non-medical equivalents, unhealthy behaviors are prevalent and medical students are at a higher risk for specific psychological ailments. Studies show that medical students are in suboptimal physical shape [8]–[12], and that they smoke [6], [13]–[15], drink [7], [16], [17], and take drugs [18], [19] like their peers in the general population. Additionally, the high risks of burnout and depression that medical students face were revealed by multiple studies [20]–[23], as well as the risk of sleep deprivation, which is not only unhealthy but also potentially hazardous [24]. Furthermore, medical students appear to eat irregularly, which might be another potential cause of fatigue, but also vice versa [25].

Additionally, if the medical students face health issues, they appear to have difficulty finding the appropriate help [26], [27]. This is partly due to a performance-driven medical culture in which work attendance as well as portraying competence and good health are demanded [28], [29], and in which the student may have trouble adapting to the patient role [30]. Embedded in medical culture is an unprofessional, inappropriate and potentially hazardous [31] practice of self-care and self-prescription. Numerous studies have shown a high prevalence of self-care, e.g., 54% of Norwegian doctors had self-prescribed over the course of a year [32], and up to 90% of Australian physicians reported that they can treat their own conditions [33]. Importantly, it seems that these practices are already acquired in medical school and increase with availability, increased clinical access and closer contact with seniors, from whom students receive ‘curb-side’ consults and drug prescriptions [27], [34].

There are no data available on these topics for Dutch medical students. We might expect them to have similar behaviors and issues as their international colleagues. Information on their health behaviors can be valuable for medical schools (and eventually, the patients) to target and prevent unhealthy habits in more effective ways, as well as to offer their students more appropriate care.

We were also interested in possible differences between senior clinically active students and junior preclinical students. The last two years of the Dutch curriculum is the clinical phase, which is commonly regarded as demanding and stressful, both physically and emotionally. Students often work over 50 hours per week, not including home study, without financial compensation. It could be that clinically active students have less healthy behaviors, as the intensive course could make students omit healthy habits or induce unhealthy coping styles. We might also expect senior students to be more tolerant towards self-prescription, as their attitudes are influenced by the clinical environment. Such effects would be valuable information for any medical curriculum comprised of a preclinical and a clinical phase. Thus, we designed this study to investigate these parameters in medical students from a major Dutch medical school. We assessed three main subjects: (1) health behaviours, (2) care needs and (3) acceptance of self-prescription, and compared answers from the preclinical and clinically active students.

## Materials and Methods

### Participants and data collection

We invited all students (first through sixth year) of a major Dutch medical school by letter and e-mail to fill out an online survey. The survey was taken in November and December 2010. The study was first approved by the school's executive board and student council, and we obtained informed consent from each participant. The medical ethical committee of the academic medical center waived the official approval procedure for this study; in the Netherlands, survey research in healthy populations does not need extensive ethical approval procedure. Participants gave their consent to participate by starting the online questionnaire. The survey took about 20 minutes to complete. It was anonymous, giving students the possibility to answer honestly. The anonymity was stressed in both the letter and e-mail.

### Survey instruments

Our survey was digital and included questions on several topics regarding health behaviors and perception, and care needs. Health behaviors were inquired in seven aspects: physical exercise, eating habits, smoking, alcohol consumption, substance use, hours of sleep per night and Body Mass Index. Care needs were investigated as a current wish for future availability of different forms of care. Acceptance of self-prescription was assessed from four angles. Specific information is provided below.

#### Demographic data

This included information about gender and study-year. A foreign descent was examined by asking “were you or were your parents born outside of the Netherlands?”, to be answered with “yes” or “no”.

#### Health behaviors

Physical exercise was measured as frequency per week and number of hours per week over the past three months, for a number of the most common sports. Time spent on sports not mentioned in our list could be entered under “Other”. Eating habits were investigated with as agreement to one of the available statements “I eat healthy”, “I eat regularly throughout the day”, “I eat my first meal before 10:00 AM” and “I eat my last meal before 8:30 PM”, each to be answered on how many days per week this is the case (0–7 times). Excessive consumption of alcohol was measured with the three item AUDIT-C screener [35]. A current smoking habit was examined with a simple “yes” or “no” question. Students were asked if they had used any tranquilizers, sleep medications or stimulants (e.g., cocaine, amphetamine, ephedrine, XTC) during the past month, each type answered with “yes” or “no”. Students' body height and weight were inquired in centimeters and kilograms. Finally, students were asked to enter the usual amount of sleep they had on working days, in hours per night.

#### Care needs

We asked students if they had a current wish for the availability of the following forms of care: physical health care, mental health care, study counseling, financial counseling, relationship counseling, help with finding housing or help making career choices, all questions to be answered with “yes” or “no”.

#### Self-prescription

We assessed students' attitudes towards self-prescription with regard to acceptability, ease, professionalism and chance of error, using four VAS scales ranging from “acceptable” (0) to “not acceptable” (100).

### Analysis

Students from study year one through four were classified as preclinical, students from study year five and six were classified as clinically active.

Each of the seven inquired health behaviors was translated to a dichotomous “healthy” or “unhealthy” variable. These behaviors were summed and classified as healthy, moderately healthy and unhealthy overall behavior.

An indicator of total physical exercise was calculated for each individual using MET-values for each listed sport, as in the compendium of MET intensities by Ainsworth et al. [36] “Other” sports were rewarded by us with a MET value of 4.5 (moderate intensity physical activity). The American College of Sports Medicine and American Heart Association guidelines recommend a minimum of 30 minutes of moderate intensity exercise (3.0–6.0 MET) for five days per week [37]. We translated this to a minimum of 450 MET-minutes per week (3.0*30*5 = 450.0). Failure to meet 450 MET-minutes per week was regarded as unhealthy behavior. Students' eating habits were judged healthy if students reported eating healthy and regularly throughout the day, measured as consuming healthy meals on at least five days per week, eating regularly throughout the day on three or more days per week, having breakfast before 10:30 on three or more days per week, and having their last meal before 20:30 on three or more days per week. On the AUDIT-C questionnaire, a cut-off score of ≥5 for males and ≥4 for females was used to indicate possible excessive drinking, and thus unhealthy behavior [35]. A current smoking habit was considered an unhealthy behavior. The Body Mass Index (BMI) was calculated based on body height and weight. A BMI outside of the normal range (<18.5 or ≥25) [38] was defined as underweight or overweight, and thus unhealthy. Any use of tranquilizers, sleep medications or stimulants was judged unhealthy. The amount of sleep needed differs widely per individual, but many studies have reported independent mortality and morbidity risks associated with both sleep less than five hours or over nine hours per night [39]. Therefore, in this study we judged a sleep pattern outside the six to eight hour range unhealthy.

These seven binary classifications of healthy or unhealthy behavior were summed to an overall individual health score. Those students without any detrimental habits were categorized as “healthy”, students with one or two unhealthy habits classified as “moderately healthy” and students with three or more unhealthy habits as “unhealthy”.

The VAS scores for the four views on self-prescription were summed to a scaled score of acceptance of self-prescription (0 = acceptable, 400 = not acceptable).

#### Statistics

Frequency of individual health scores in the preclinical and clinically active population were tested with a chi-squared test for trend (Kendall's tau-b). Differences in care needs per subpopulation were tested using chi-squared tests. The difference in acceptance of self-prescription between the groups was investigated using a Student's independent t-test. The number of cases in the analyses varied because of incidental missing values. All tests were two-tailed and *p*<0.05 was considered significant.

## Results

A total of 954 students filled out the questionnaire (relative response rate = 35%). Data of 902 students could be used for this study as some incidental questions were left blank and only complete individual sets were used. Student demographics (Table 1) did not differ significantly between preclinical and clinical students, with male sex of 33% vs. 30% (*p* = 0.37) and Dutch natives 76% vs. 79% (*p* = 0.21). Percentages of 30–35% male and ca. 33% clinically active participants correspond with the actual ratio of sexes and build of the curriculum of the course.

**Table 1:** Demographics of the student population.

Demographics.	Total	Preclinical	Clinically active
*n*	902	586	316
GenderMale	286 (31.7%)	192(32.8%)	94(29.7%)
Female	616(68.3%)	394(67.2%)	222(70.3%)
EthnicityNative	694(76.9%)	443(75.6%)	251(79.4%)
Foreign	208(23.1%)	143(24.4%)	65(20.6%)

(The distribution of respondents reflects the structure of the curriculum and gender distribution of the population.)There were no significant differences between preclinical and clinical students.

In total, 9% of students did not meet the minimum amount of MET-minutes per week, and 6% of students had a current smoking habit. Unhealthy eating habits were prevalent with 51% of students not meeting the criteria set. Additionally, unhealthy alcohol consumption was prevalent with 46% of students meeting the AUDIT-C criteria for risk of alcohol-related health problems. Underweight or overweight was present in 20% of students. Health behaviors are shown in Table 2.

**Table 2:** The prevalence of unhealthy habits in the total student population (n = 902) and in the preclinical (n = 586) and clinical (n = 316) subgroup.

Health behaviors.Numbers as percentages of the subpopulation	Physical activity<450 MET-min/week%	Unhealthy eating habits%	Alcohol at risk score%	Smoking%	Substance use%	Over- or underweight%	Abnormal sleep pattern%
Preclinical	8.3	53.3	47.8	6.5	5.2	18.7	8.7
Clinically active	9.3	46.7	41.4	6.2	7.9	21.3	5.0
Total	8.6	50.9	45.6	6.4	6.2	19.6	7.4

The classification in healthy, moderately healthy and unhealthy categories is shown in Table 3. There were no significant differences in these categories between the preclinical and clinical group (*p* = 0.54), with 17% of preclinical and 20% of clinical students reported not to have any unhealthy behaviors.

**Table 3:** Classification of students based on summed health score.

Health classification.Numbers as percentages of the subpopulation	n	Healthy(0 unhealthy behaviors)%	Moderately healthy(1–2 unhealthy behaviors)%	Unhealthy (>2 unhealthy behaviors)%
Preclinical	586	17.0	68.3	14.7
Clinically active	316	19.7	65.3	15.0
Total	902	18.0	67.2	14.8

No significant differences (*p* = 0.54).

The forms of care most needed were help with career choices, physical health care, help with study problems and mental health care. In preclinical students, the need for physical health care, help with study problems, help with career choices and help with financial problems was significantly higher (*p*<0.05), as can be seen in Table 4.

**Table 4:** Students' need for availability of different forms of care as a percentage of the total population and of the preclinical and clinical subgroup.

Care needs.Numbers as percentages of the subpopulation	Mental health care%	Physical health care%	Help with study problems%	Help with career choices%	Help with financial problems%	Help with relational problems%	Help with housing problems%
Preclinical	41.8	55.5*	55.2*	74.0*	24.1*	13.4	25.1
Clinically active	41.8	40.8*	35.9*	63.8*	16.4*	9.2	20.4
Total	41.8	50.3	48.3	70.4	21.4	11.9	23.5

*difference between preclinical and clinically active subgroup = *p*<0.05.

Finally, the summed score of acceptance of self-prescription was significantly higher in the clinical subgroup over the preclinical subgroup (181±4 vs. 142±5 *p*<0.001). The scales for acceptability of self-prescription were tested for internal reliability using Cronbach's alpha and were found reliable at 0.83.

## Discussion

Overall, the data gives an equivocal message on the healthiness of the students. It is satisfying that over 90% of students appear to get frequent and sufficient physical exercise, and over 80% of students have a BMI within the normal range. However, their eating habits are predominantly unhealthy and/or irregular. Smoking prevalence is relatively low, as are abnormal amounts of sleep per night. It is certainly worrying that almost half of the students meet the AUDIT-C criteria for risk of alcohol-related health problems, and over 6% used stimulants, tranquilizers or sleep medications in the past 30 days.

Contrary to expectations, the demands of the clinical phase of the curriculum appear to have no significant impact on the health behaviors of the students.

The form of care most needed was help with career choices. Students also expressed high needs for easily accessible mental health care, physical health care and help with study problems. The need for physical health care is significantly lower in the clinical phase, whereas the need for mental health care was equally high. The need for help with study problems, career choices and financial problems was also significantly lower. Among students from the clinically active subgroup, self-prescription was considered more acceptable compared to their preclinical colleagues.

The strength of this study is the large absolute number of participants. However, the relative response rate is somewhat low. This may be explained by the fact that, unlike other studies, participation in this study was completely voluntary. Although we are aware of the potential source of bias that the non-response may initiate, we have no reason to suspect the participants not to be representative of the total population because of the comparable numbers of participants from every study year and the variation in the observed responses. Still, it could be that unhealthy behavior is underreported, as is often the case with this kind of research. These findings then reflect too favorable results. Another strength is that, to our knowledge, the curriculum of the investigated medical school hardly changed over the past years, so that found differences are unlikely caused by changes in education or services offered.

Eating habits of the students were mostly irregular and/or unhealthy, as was alcohol consumption. Perhaps this is to be expected from young college students moving out from their parents. Both behaviors were less prevalent among senior clinically active students.

Need for help with study problems, financial problems and career choices was significantly lower among senior clinically active students. Perhaps this can also be ascribed to student maturation and growing competence. Interestingly, a significantly lower need for physical health care was found, whereas the need for mental health care was equally high. Roberts et al. and Hooper et al. already reported senior students to have more ‘curb-side’ consults from colleagues, seniors or themselves, which could explain the lower care needs [27], [34]. As these informal consults are more adequate for physical than mental health problems, and given the greater stigma surrounding mental health issues, mental health curb-side consults are probably obtained less. Indeed, Roberts et al. also reported student fear of academic jeopardy to be much higher with mental health problems [30].

The higher acceptance of self-prescription we found in clinically active students in our study could reflect students' growing confidence in their own medical skills, yet also suggests that exposure to the clinical medical culture has a role herein. The higher acceptance worries us as this could mean that senior students will seek less formal consultation and have a growing tendency to manage themselves. Finally, not shown above but worth mentioning is that the use of sleep medications was three times as high among clinically active students (1.8% vs. 5.6% P = 0.004). The higher use of sleep medications could be indicative of sleeping problems due to nights shifts, but also higher amounts of stress in this stage of the training.

By comparing this study's results with other figures, the prevalence of over or underweight (20%) among our medical students was lower than in the general population [40], Greece and Malaysia [11], [12], but higher than Colombia [10]. Smoking prevalence (6%) was also lower than in the general population [40], comparable to medical students from the US [6], [9] and lower than medical students from Colombia, Vietnam and Poland [10], [13], [14].

The results indicate that over eighty percent of medical students, future promoters of public health, act against their own physical wellbeing in at least one of the ways investigated. In particular, the high rates of alcohol abuse are troubling, which makes it incomprehensible that this is not at all addressed in medical school. When alcohol abuse is learned young, students could well develop a disposition for dependence; rates of alcohol and/or drug dependence among doctors are reported to be as high as 9–12% [18], [41].

The need for specific forms of care is high, which is interesting for faculties to meet their students' needs. If these forms of care are offered and easily accessible, this could contribute to an environment in which help-seeking behavior is normal and encouraged, and could serve as an alternative to curb-side consultations, self-care and self-prescription. Senior students growing towards higher acceptance of self-prescription can be seen as a growing mandate to offer them an appropriate and comfortably accessible alternative. For physical health care, a school physician could provide in this need, whereas help with career choices, mental health care and study problems could be met by a school psychologist.

Importantly, greater school support for health promotion was already found positively associated with a higher counseling frequency [42]. Potential supportive measures would be more education on smoking, alcohol and drugs [43] physical activity programmes [8] and a programme teaching students the basic of preparing healthy food [44]. An integrated lifestyle curriculum could deliver all these components at once, and is highly recommended. An valuable example of such a course was recently designed in the United Arab Emirates [45]. As our findings suggest that there is no significant deterioration of health behaviors over the years of medical training, life-style changes achieved in the first year of medical school might last throughout the entire curriculum. It can be concluded that there is ample room for improvement of health behaviors of medical students. In the light of evidence, medical faculties have a responsibility in pro-actively promoting health of their students and eliminating the barriers that stand in the students' way to appropriate care. If done so, it is likely that the benefit of healthier and happier medical students will be measurable in a higher wellbeing of the physicians and better preventive care for their patients, and therefore better health for all.
